# Atopic dermatitis-related anti-inflammatory in vitro effects of a plant extract mixture

**DOI:** 10.1038/s41598-025-09053-4

**Published:** 2025-07-03

**Authors:** Nina Heinemann, Franziska Rademacher, Henning Vollert, Regine Gläser, Jürgen Harder

**Affiliations:** 1 Department of Dermatology Quincke Research Center, Kiel University, Rosalind-Franklin Str. 9, Kiel, 24105 Germany; 2 Bioactive Food GmbH, Bad Segeberg, Germany

**Keywords:** Atopic dermatitis, Aryl hydrocarbon receptor, S. aureus, Skin inflammation, Antioxidant, Plant extract mixture, Skin diseases, Infection, Chronic inflammation, Translational research

## Abstract

The chronic inflammatory skin disease atopic dermatitis (AD) is characterized by barrier defects, inflammation and microbial dysbiosis with a high prevalence of *Staphylococcus (S.) aureus*. Previously, we reported that a plant extract mixture consisting of curly kale, green tea and apple had beneficial effects on AD. Herein we further investigated the underlying promising effects exerted by the plant extract. The extract exhibited direct antimicrobial activity against *S. aureus* and reduced the number of extra- and intracellular *S. aureus* in keratinocytes. Gene expression analyses and luciferase reporter assays in 2D and 3D AD-like skin models showed activation of the aryl hydrocarbon receptor (AhR) by the plant extract. Knockdown experiments with siRNA revealed an AhR-dependent induction of the skin barrier molecule filaggrin by the plant extract in the AD skin model. Moreover, the plant extract demonstrated antioxidative properties by reduction of intracellular reactive oxygen species and induction of the antioxidative enzyme NAD(P)H quinone oxidoreductase 1 (NQO1). In conclusion, the antioxidant and antibacterial effects of the plant extract in combination with the AhR activation may be promising for the topical treatment of AD. Further research has to uncover the full potential of plant extracts for the treatment of AD and other inflammatory diseases.

**Supplementary Information:**

The online version contains supplementary material available at 10.1038/s41598-025-09053-4.

## Introduction

Atopic dermatitis (AD) is a chronic inflammatory skin disease which is characterized by recurrent localized itchy eczema^1^. The mechanisms underlying AD are still not fully understood, because of its complex etiology. Genetic susceptibility and environmental trigger factors are discussed to induce skin barrier defects and T-helper type (Th)2-driven immune dysregulation^1^. Additionally, a microbial dysbiosis with high prevalence of *Staphylococcus (S.) aureus* is seen in AD lesions, which contributes to inflammation and worsens skin barrier defects^2,3^. Density of *S. aureus* on skin correlates with disease severity in AD patients^4^.

Interaction between inflammation and barrier disruption results in chronic inflammation, which is accompanied by an overproduction of reactive oxygen species (ROS). Many studies show that in AD ROS production is induced while the antioxidant capacity is reduced^5^. For example, Koch and colleagues showed that pathway activation mediated by the antioxidant nuclear factor erythroid-2-related factor (Nrf) 2 is reduced in AD skin^6^. Nrf2 is a transcription factor that regulates the expression of antioxidant enzymes and plays a crucial role in eliminating ROS^7,8^. This imbalance hampers an adequate elimination of reactive oxygen species by the antioxidant system and oxidative stress occurs: free radicals such as superoxide anions (O_2_^−^) and hydrogen peroxide (H_2_O_2_) cause oxidative damage to different macromolecules in the cell which again contributes to inflammation^9^.

Due to the complexity of AD, medical treatment is challenging. At least, with the development of monoclonal antibodies that block signaling of Interleukin (IL)−4 or IL-13 and Janus kinase (JAK) inhibitors, treatment options for moderate-to-severe AD have clearly improved in the past years^10–12^. However, for mild-to-moderate AD, topical treatment with emollients and immunosuppressants (corticosteroids and calcineurin-inhibitors) is still the standard treatment^13^. Because long-term use of these immunosuppressants may have adverse effects^14^ there is still a need for novel topical therapeutics.

A promising new target for topical AD treatment is the aryl hydrocarbon receptor (AhR), a transcription factor, which can be activated by a variety of different exogenous or endogenous ligands. Effects of AhR activation are strongly ligand-depending and cell-specific^15^. It is known that activation of the AhR in skin by physiological ligands leads to epidermal differentiation by upregulation of barrier molecules and has impact on inflammation^16–18^. Certain AhR ligands may therefore be presumed to be beneficial in cutaneous inflammatory diseases with barrier defects. In fact, the AhR modulator tapinarof (3,5-dihydroxy-4-isopropyl-trans-stilbene) actually approved for psoriasis treatment from the “food and drug administration” (FDA), showed beneficial effects in AD^17,19,20^.

The effect of tapinarof is also attributed to its antioxidant activity mediated by the transcription factor Nrf2, which is closely linked to the AhR pathway^17,21,22^. Besides tapinarof, other dual AhR/Nrf2 activating modulators with antioxidant effects such as coal tar or soybean glyteer have already shown beneficial effects on AD^16,23,24^. Additionally, many phytochemicals are discussed to have AhR and Nrf2 modulating properties, too^25^. Moreover, a lot of plant metabolites show antibacterial effects, also against *S. aureus*^26,27^. A combination of antibacterial effects against *S. aureus* and AhR modulation resulting in antioxidant activity may be especially promising for AD treatment. Plant extracts seem to be a good resource for such a compound due to the high diversity of bioactive metabolites.

Recently, we have examined a specific plant extract mixture for its effects on AD. This plant extract showed beneficial effects in AD patients in a clinical cohort study. Signs of inflammation (local “Scoring Atopic Dermatitis Index“ SCORAD) and transepidermal water loss were lowered and the itch was reduced^28^. In line with that, gene expression analysis of a two-dimensional (2D) AD skin model showed an upregulation of skin barrier molecules such as filaggrin and loricrin by the plant extract and downregulation of AD typical inflammatory markers such as carbonic anhydrase 2 (CA2), CC-chemokine ligand (CCL)26 and IL-24^28^. Now, the primary focus of this study is to determine the underlying mechanisms responsible for the observed beneficial effects with main focus on the role that AhR may play in this process. Additionally, the antioxidant capability of the plant extract was examined and we sought to investigate whether the extract exhibits antibacterial effects.

## Results

### Plant extract showed antibacterial effects against different *S. aureus* strains

**Fig. 1:**
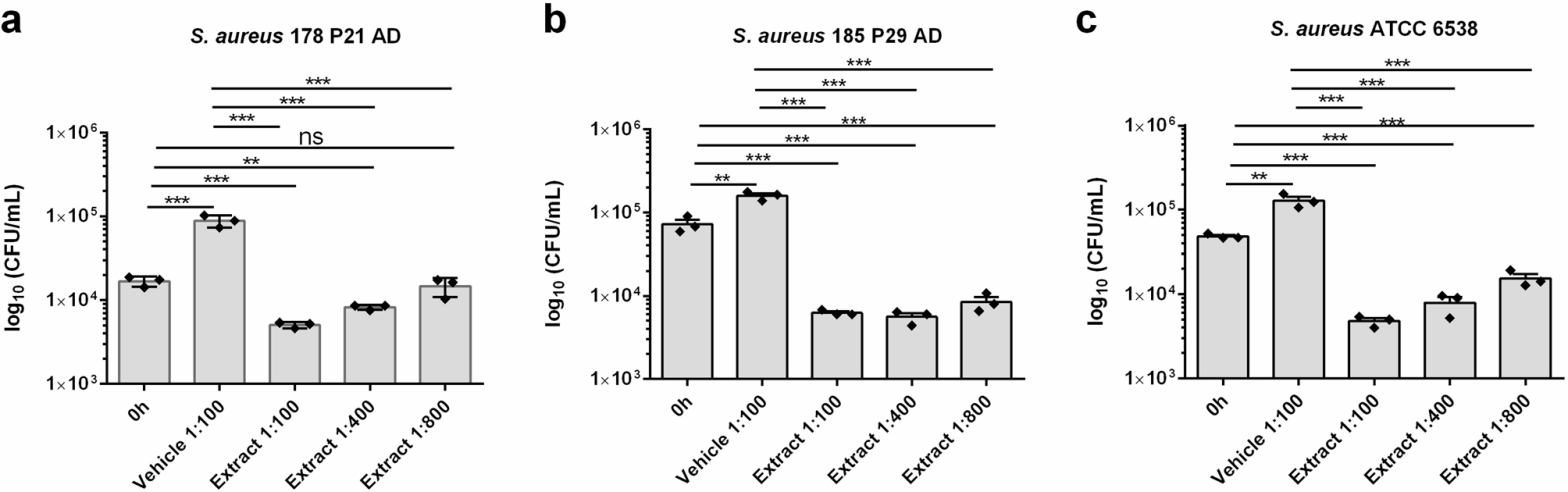
Plant extract showed antibacterial effects against different *S. aureus* strains. Two *S. aureus* strains isolated from AD skin (**a**,**b**) and one ATCC strain (**c**) were exposed to different concentrations of the plant extract for 2 h. Bacteria were plated on TSB agar and the colony forming units (CFU) were counted after 24 h. Medium with vehicle (5% EtOH) served as control. CFU values are shown as log₁₀-transformed data. Statistical analyses were performed on log-transformed values. Bars are means ± SEM. Statistical significance was determined using One-way ANOVA with subsequent Bonferroni’s Multiple Comparison Test (*n* = 3, **p < 0.01, ***p < 0.001; ns = not significant).

To determine if the plant extract has antibacterial effects, two *S. aureus* strains isolated from lesional skin of AD patients and one ATCC strain were exposed to different concentrations of the plant extract for 2 h. The plant extract inhibited the growth of all three *S. aureus* strains dose-dependently as the CFUs after 2 h were lower compared to the CFUs of the bacteria treated with vehicle control only. In most cases, treatment with the plant extract even resulted in CFUs lower than at the starting point (0 h), demonstrating bactericidal effects (Fig. 1).

### Plant extract reduced number of extra- and intracellular *S. aureus* in NHEKs

**Fig. 2:**
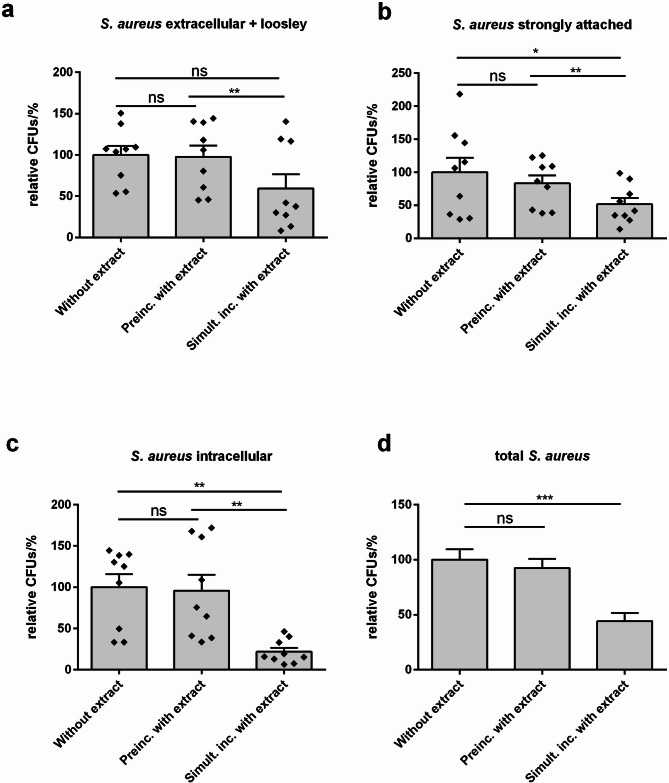
Plant Extract reduced number of extra- and intracellular *S. aureus* in NHEKs. NHEKs were treated for 2 h with *S. aureus* (strain 178 P21) with and without the plant extract. NHEKs were also preincubated for 24 h with the plant extract for determination of preventive effects. After 2 h (**a**) extracellular (**b**) strongly attached and (**c**) intracellular bacteria were plated on TSB-agar and CFUs were counted after incubation for 24 h at 37 °C. (**d**) For determination of total CFUs, the number of extracellular, strongly attached and intracellular bacteria were combined (*n* = 27). For a better comparison, the values for stimulation without extract in the different experiments were set as 100%. Bars indicate mean + SEM. Statistical significance was determined using One-way ANOVA with subsequent Bonferroni’s Multiple Comparison Test (*n* = 9, *p < 0.05, **p < 0.01, ***p < 0.001; ns = not significant).

Besides this direct antibacterial effect, it was also tested if the plant extract may hinder the adherence of *S. aureus* to primary normal human epidermal keratinocytes (NHEKs) or the number of intracellular *S. aureus* in NHEKs. Therefore, NHEKs were treated with living AD-derived *S. aureus* (*S. aureus* strain 178 P21) with and without the extract mixture for 2 h. Additionally, NHEKs were preincubated with the plant extract before infection with *S. aureus* to determine possible preventive effects. After 2 h, extracellular, strongly adherent or intracellular bacteria were plated on TSB agar plates and CFU were counted after overnight incubation. Simultaneous treatment with *S. aureus* and the plant extract reduced the number of bacteria at all locations in comparison to treatment without plant extract (Fig. 2a-c). Especially the number of intracellular bacteria was strongly reduced by almost 80% (Fig. 2c). The total number of bacteria after 2 h was significantly reduced by half when applying *S. aureus* and extract simultaneously (Fig. 2d). Preincubation with the plant extract had no significant effects on *S. aureus* (Fig. 2). Moreover, the plant extract did not induce the principal *S. aureus* killing antimicrobial peptides (AMPs) human beta defensin (hBD) 3 and ribonuclease (RNase) 7 in NHEKs (Supplementary Figure S1)^29–32^.

### Preincubation with plant extract reduced inflammation induced by *S. aureus* in NHEKs

**Fig. 3:**
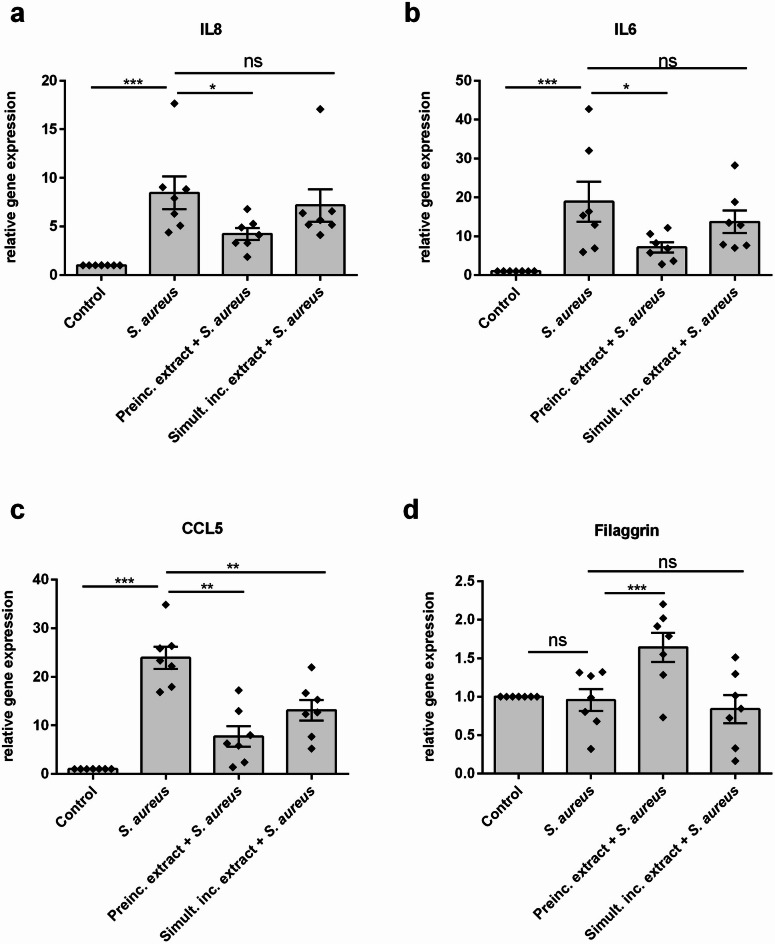
Preincubation with Plant Extract Reduced Inflammation Induced by *S. aureus*. NHEKs were preincubated with vehicle for 24 h and then treated with the bacteria for 3 h in absence (*S. aureus*) or in presence of the extract (Simult. Inc. extract + *S. aureus*). NHEKs were also preincubated with the plant extract for 24 h and then treated with the bacteria in absence of the plant extract for 3 h (Preinc. Extract + *S. aureus*). Then, gentamycin was added and incubation was continued for 24 h. Gene expression levels of (**a**) IL-8, (**b**) IL-6 (**c**) CCL5 and (**d**) filaggrin were determined by real-time PCR. Statistical significance was tested by One-way ANOVA test with subsequent Bonferroni’s multiple comparison test. Bars are means  ± SEM of seven experiments performed in triplicates (*n* = 7; *p < 0.05; **p < 0.01; ***p < 0.001; ns = not significant).

In addition to antibacterial effects, many plant components like flavonoids exhibit anti-inflammatory effects. Because colonization of skin with *S. aureus* leads to an inflammatory response in keratinocytes, it was tested if the plant extract attenuated also *S. aureus*-induced inflammation. Therefore, NHEKs were either preincubated with the plant extract for 24 h and then treated with *S. aureus* strains SA 178 P21 (Fig. 3) or SA 185 P29 (Supplementary Figure S2) or NHEKs were simultaneously incubated with the extract only during *S. aureus* infection. As a result, stimulation with *S. aureus* alone led to an increased gene expression of the inflammatory markers IL-8, IL-6 and CCL5 (Fig. 3a-c). Interestingly, preincubation with the extract mixture led to a reduced gene expression of these inflammatory markers, while simultaneous incubation of extract and *S. aureus* had weaker effects and reduced the expression of only CCL5 (Fig. 3c). Additionally, the extract preincubation induced a significant higher gene expression of the skin barrier molecule filaggrin (*FLG*) compared to *S. aureus-*treated cells only (Fig. 3). Simultaneous incubation of extract and *S. aureus* had no impact on the gene expression of *FLG*.

### Plant extract activated the Aryl hydrocarbon receptor (AhR)

**Fig. 4:**
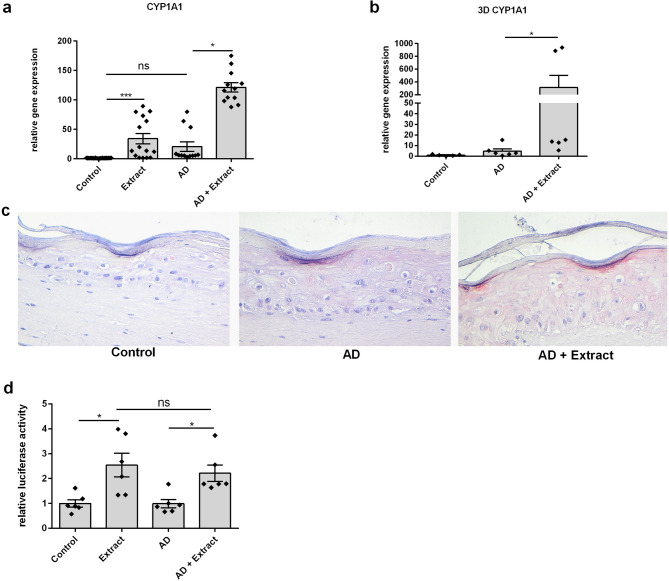
Plant Extract Activated Aryl Hydrocarbon Receptor. (**a**) CaCl_2_-differentiated NHEKs were left unstimulated (Control) or stimulated with the plant extract (Extract) for 20 h. (**a**) NHEKs or (**b**) a 3D skin model was also treated for 20 h with an AD-like cytokine mixture (IL-13, IL-4, IL-22 and TNF-alpha, each 10 ng/mL) either in the absence (AD) or in the presence of the plant extract (AD + Extract). Gene expression levels of CYP1A1 were determined by real-time PCR. Statistical significance was tested by (**a**) One-way ANOVA test with subsequent Bonferroni’s multiple comparison test (*n* = 12) or (**b**) Mann-Whitney test (*n* = 6; *p < 0.05; **p < 0.01;***p < 0.001; ns = not significant). (**c**) Detection of CYP1A1 in the 3D skin by immunohistochemical staining. (**d**) For determination of AhR activation, cells were transfected with the pGudLuc6.1 reporter plasmid in which the *firefly* luciferase gene expression is dependent on AhR activation, and the pGL4.74 plasmid (reference control plasmid containing *renilla* luciferase). One day after transfection cells were stimulated as described in (**a**). Activation was determined by analyzing relative luciferase activity. Statistical significance was tested by Kruskal-Wallis test with subsequent Dunn’s multiple comparison (*n* = 6; *p < 0.05, ns = not significant). Bars indicate means + SEM.

As described in the introduction, we recently reported an upregulation of skin barrier molecules such as filaggrin and loricrin and a downregulation of AD typical inflammatory markers such as CA2, CCL26 and IL-24 in a 2D AD skin model by the plant extract^28^. In skin, the aryl hydrocarbon receptor (AhR) is important for barrier function and has an impact on inflammation^33^. Thus, we evaluated if the plant extract is able to activate the AhR and if this activation is responsible for the beneficial effects seen in the 2D AD model. To this end, we analyzed expression of the gene *CYP1A1*. *CYP1A1* is a direct response gene of the aryl hydrocarbon receptor and is induced upon activation of the AhR^34^. As a result, the plant extract induced gene expression of *CYP1A1* in a 2D in vitro model (Fig. 4a) and a 3D skin model (Fig. 4b). Immunohistochemical staining detected higher levels of CYP1A1 in the 3D skin model that was stimulated with the plant extract and AD cytokines than in the control or the AD-stimulated skin only (Fig. 4c). Together, these data suggest that the plant extract activates the AhR. To further evaluate AhR activation by the plant extract, keratinocytes were transfected with the pGudLuc6.1 AhR reporter plasmid in which the *firefly* luciferase gene expression is dependent on AhR activation. Stimulation of the transfected NHEKs with the plant extract increased luciferase activity in untreated keratinocytes or the 2D AD model indicating activation of the AhR (Fig. 4d).

### Impact of AhR KD on effects of the plant extract in the AD model

**Fig. 5:**
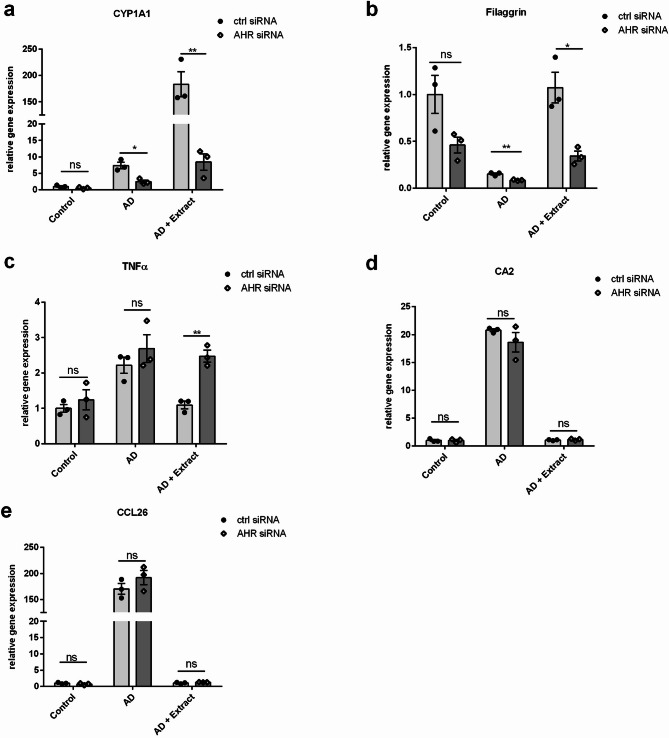
Impact of AhR siRNA-Mediated Knockdown on the Effects of the Plant Extract in the 2D AD Model. NHEKs were transfected with AhR siRNA or control siRNA. 48 h later, NHEKs were stimulated with the AD-associated cytokine mixture (IL-13, IL-4, IL-22 and TNF-alpha, each 10 ng/mL) either in the absence (AD) or in the presence of the plant extract (AD + Extract). Gene expression levels of (**a**) CYP1A1 (**b**) filaggrin (**c**) TNFα (**d**) CA2 and (**e**) CCL26 were determined by real-time PCR. Statistical significance was tested by unpaired t-test (*n* = 3; *p < 0.05; **p < 0.01; ns = not significant). Bars indicate means ± SEM.

Next, we sought to determine if the AhR activation by the plant extract is responsible for the previously reported beneficial effects in the AD model^28^. Therefore, an AhR knockdown (KD) model was used, in which the expression of AhR was reduced via AhR siRNA (KD efficiency ≈ 60%, Supplementary Figure S3) in the 2D AD model. Transfection with control siRNA served as control. In this AhR KD model and in the control model without AhR KD, the incubation with AD cytokines reduced gene expression of filaggrin and induced gene expression of TNFα, CA2 and CCL26. When adding the plant extract to the AD model without AhR KD, a strong CYP1A1 induction was measured (Fig. 5a). In line with our previous results, the plant extract restored the downregulated filaggrin gene expression (Fig. 5b) and reduced the gene expression of the inflammatory markers TNFα, CA2 and CCL26 (Fig. 5c-e). In contrast, the effects of the extract in the AhR KD model partly differed from these results: As expected, the AhR KD reduced the plant extract-mediated induction of the AhR downstream target CYP1A1 (Fig. 5a). In line with that, the induction of filaggrin gene expression by the extract was decreased in the AhR KD model (Fig. 5b). Additionally, downregulation of TNFα by the extract was no longer observed upon AhR KD (Fig. 5c). In contrast, the AhR KD did not influence the plant extract-mediated downregulation of CA2 and CCL26 (Fig. 5d, e). We obtained consistent results when inhibiting the AhR via AhR inhibitor CH-223191 (Supplementary Figure S4).

### Plant extract induced gene expression of the antioxidant enzyme NQO1

**Fig. 6:**
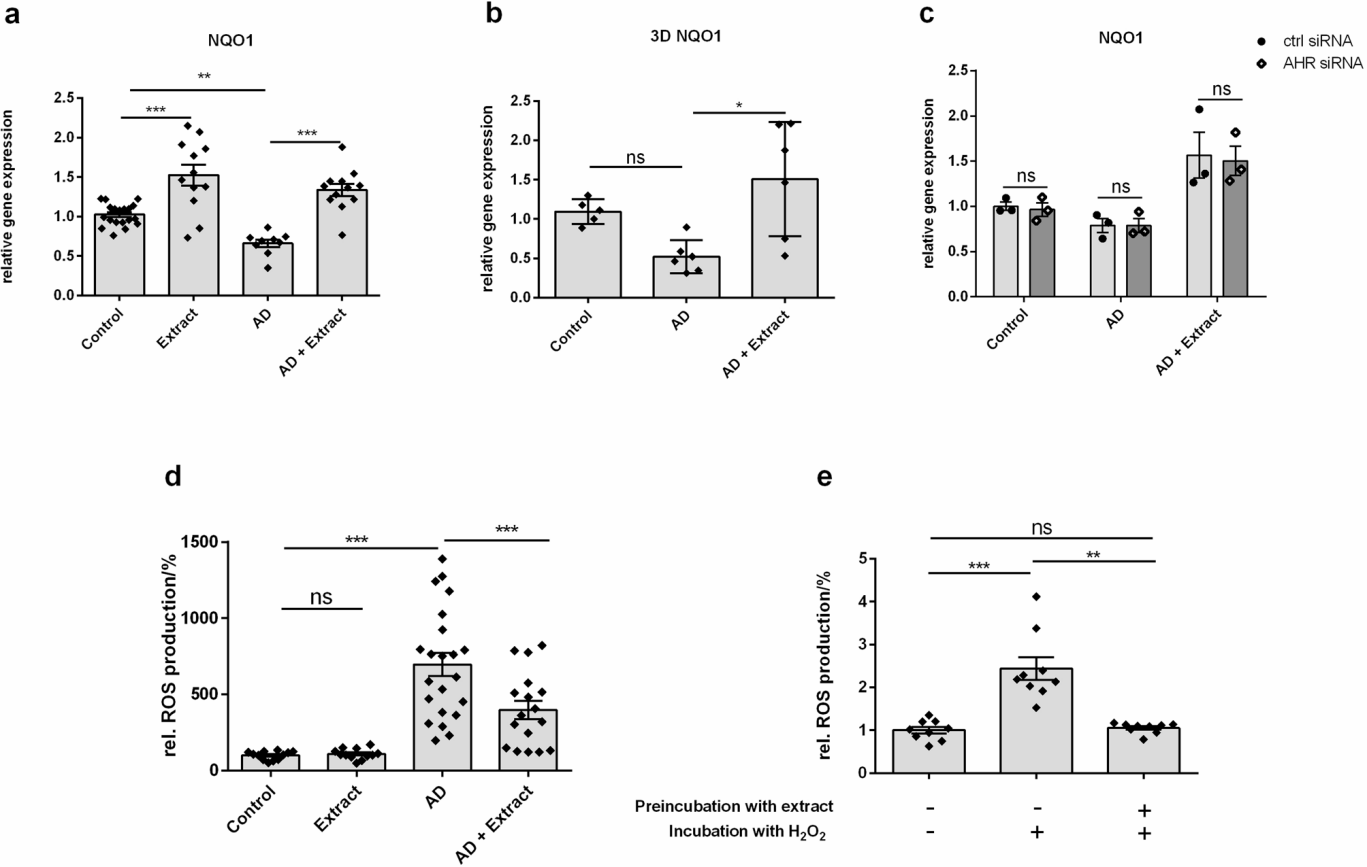
Expression of the Antioxidant Enzyme NQO1 is Induced and Intracellular ROS Production is Reduced by the Plant Extract. (**a**) NHEKs and (**b**) 3D skin models were stimulated as described in Fig. 4 or (**c**) Fig. 5. Gene expression levels of NQO1 were determined by real-time PCR. Statistical significance was tested by (**a**) One-way ANOVA test with subsequent Bonferroni’s multiple comparison test (*n* = 12) or (**b**) by Kruskal-Wallis test with subsequent Dunn’s multiple comparison test (*n* = 6) or (**c**) by unpaired t-test (*n* = 3; *p < 0.05; **p < 0.01; ***p < 0.001, ns = not significant). (**d**, **e**) Intracellular ROS were determined by using DCFDA Cellular ROS Detection Assay (Abcam). (**d**) NHEKs were stimulated as described in Fig. 4. After stimulation, intracellular ROS were measured. (**e**) NHEKs were stimulated with the extract for 24 h. Then, cells were washed and treated with 250 µM H_2_O_2_ for 10 min. After stimulation, intracellular ROS were measured. Statistical significance was tested by Kruskal-Wallis test with subsequent Dunn’s multiple comparison test (**d**) *n* = 12; (**e**) *n* = 9;  **p < 0.01, ***p < 0.001, ns = not significant). Bars indicate means ± SEM.

AhR activation can also regulate the antioxidant response in cells by activating the Nrf2 pathway. To analyze the activation of the Nrf2 pathway, gene expression of the antioxidant enzyme NQO1, a Nrf2 downstream target, was measured in the 2D AD model (Fig. 6a) and the 3D AD model (Fig. 6b). Interestingly, the NQO1 expression was slightly downregulated in both AD models. Stimulation with the plant extract significantly induced NQO1 expression in both models again. Even stimulation with extract alone induced NQO1 gene expression in the 2D model. The induction of NQO1 was not affected by AhR KD (Fig. 6c) suggesting an AhR-independent induction of NQO1 by the plant extract.

### Plant extract reduced intracellular reactive oxygen species

To test if the plant extract increases the intracellular antioxidative potential, the intracellular ROS level was measured in the 2D AD model via a fluorometric ROS detection assay (DCFDA assay). Treatment with AD cytokines led to more than 6 times higher intracellular ROS production in comparison to the untreated control. Adding the extract to the AD model reduced the amount of ROS by half (Fig. 6d) confirming the antioxidant potential of the extract. Stimulation with the extract alone did not show any differences from the control.

In order to test if the extract also shows preventive antioxidant effects against ROS, NHEKs were preincubated with or without the extract for 24 h. Then cells were washed and fresh medium with 250 µM H_2_O_2_ as a stressor was added. As shown in Fig. 6e preincubation with the extract prevented the cells from ROS production suggesting that cellular antioxidant pathways had been activated by the extract leading to a higher antioxidant potential of the cells and a preventive effect against trigger factors such as H_2_O_2_.

## Discussion

Our study shows that the plant extract activates the AhR and exhibits combined antibacterial, antioxidant and anti-inflammatory properties in 2D- and 3D organotypic AD-like skin models. These characteristics may be in part responsible for the beneficial effects of the plant extracts observed in a recent pilot study with AD patients^28^.

The antibacterial properties were seen by a dose-dependent inhibition of the growth of *S. aureus* by the plant extract. This confirms that compounds in the extract mixture act directly on *S. aureus*, whereby the exact mechanisms remain unclear. In general, the most common antibacterial mechanism of phytochemicals is the disruption of plasma membrane structure, for example by pore formation, altering polarity or increasing permeability^35^. In plant extracts, the antimicrobial activity is strongly connected with the content of phenolic components due to their structure-depending interaction with lipid bilayers such as the membrane^35^. For example, in green tea extract, the most abundant polyphenol epigallocatechin-3-gallate (EGCG) demonstrates strong antimicrobial activity against *S. aureus* by binding to lipid layers and peptidoglycans, which leads to disruption of the cell membrane^36^.

Some natural molecules also have indirect antibacterial effects by inducing the production of antimicrobial peptides (AMPs) in target cells^37,38^. We did not observe a significant induction of the principal *S. aureus*-killing AMPs hBD-3 and RNase 7 in NHEKs by the extract indicating that the extract alone does not induce preventive antibacterial activity against *S. aureus*. In line with this, we observed antibacterial effects only by simultaneous treatment of NHEKs with plant extract and *S. aureus*, while preincubation of NHEKs with the plant extract did not affect the number of living bacteria. The observed strong reduction of intracellular *S. aureus* in NHEKs treated simultaneously with *S. aureus* and extract may be therefore a result of direct bactericidal effects of the plant extract. A weakening of the bacteria could also play a role, making it more difficult to infect the target cell. For example, damage of cell membrane by EGCG quickly changes the gene expression of *S. aureus* to a repair mode which induces the expression of genes involved in membrane transport, but inhibits the gene expression of fibrinogen-binding proteins, which are important for adhesion and invasion of host cells^39,40^.

Although preincubation with the extract had no effects on antibacterial activity, we could show that preincubation with the extract decreased *S. aureus*-induced expression of inflammatory mediators in NHEKs. For the anti-inflammatory effects, preincubation seems to be necessary, as the simultaneous stimulation of *S. aureus* treated NHEKs with the extract had markedly weaker anti-inflammatory effects. The *S. aureus-*induced upregulation of inflammation markers in our in vitro NHEK model is consistent with the observation of others^41^ and it is known that *S. aureus* expresses a lot of different soluble or cell wall anchored virulence factors which contribute to inflammation in skin^42^. As mainly the preincubation with the extract had anti-inflammatory effects, it seems likely that the observed preventive anti-inflammatory effect of the extract can be explained by the modulation of signaling pathways in NHEKs. We could show that the extract activates the AhR pathway, thus the AhR activation by the extract may be in part responsible for the anti-inflammatory effects seen here. The observed induction of filaggrin gene expression by preincubation with the extract may also depend on AhR activation as AhR activation is known to induce the expression of skin barrier molecules^43^. Interestingly, Brauweiber and colleagues reported that filaggrin expression can protect cells against alpha toxin damages by mediating the secretion of sphingomylinase, an enzyme which reduces the number of alpha toxin binding sites on keratinocytes’ surface^44^. Alpha toxin is a well-known soluble factor of *S. aureus*, which increases cytokine production in target cells. It would be interesting to investigate whether the reported effects against *S. aureus*-induced inflammation are partly due to the upregulation of filaggrin expression by the plant extract.

The AhR activation may also be responsible for the beneficial effects seen in the 2D in vitro AD model, as numerous other AhR ligands described in the literature have positive effects on AD. In these reports the AhR activation strengthened the skin barrier and reduced inflammation, both important aspects in the treatment of AD^16,17,20,24^. Inflammation in AD is Th2 driven and Th2 cytokines increase the expression of the typical AD markers CCL26, an eosinophil recruiting chemokine, and CA2^45^, while they decrease the expression of skin barrier molecules such as filaggrin^46^. Our in vitro AD model mimics these conditions well. The Th2 effects are mainly mediated via the signal transducer and activator of transcription (STAT)3 and STAT6 signaling^47^. The positive effects of many AhR agonists on AD are connected with an inhibition of the phosphorylation of STAT6^16,48,49^ or a competition on DNA binding between STAT6 and AhR^18^. It was reported that AhR activation inhibits the phosphorylation of STAT6 and thus restores the Th2-mediated downregulation of skin barrier molecules such as filaggrin^16,50^. Our experiments revealed that the extract restored filaggrin expression in an AhR-dependent way, too. Further experiments have to determine whether this AhR-dependent filaggrin induction is mediated via STAT6 modulation.

Regarding the inflammation markers in the AD model, we observed an AhR-dependent inhibitory effect of the extract on TNFα expression, whereas the downregulation of CCL26 and CA2 was AhR-independent. This is in contrast to the results of Alangari and colleagues, who reported an AhR-dependent CCL26 downregulation in an AD model by manuka honey^51^. The different results may be explained by the different composition of manuka honey and the plant extract: both contain a variety of different compounds making it difficult to directly compare the effects of the extracts.

Another anti-inflammatory mechanism of the plant extract may be related to antioxidant properties. For example, Dwivedi and colleagues reported that reduced ROS levels decreased IL-4-induced phosphorylation of STAT6 in T cells^52^. Additionally, it was reported for tapinarof and other AhR agonists with beneficial effects for AD, that they contain antioxidant properties^50^. Besides the previously shown free radical scavenging activity of the plant extract^28^we further hypothesized that the plant extract also exhibits intracellular antioxidant effects in keratinocytes. In line with this hypothesis, intracellular ROS were reduced when incubating the AD in vitro models simultaneously with the extract mixture. The extract mixture alone did not lead to an increased intracellular ROS, thus confirming that the AhR activation with its induced high CYP1A1 gene expression does not lead to oxidative damage by the plant extract. This is in contrast to the effects of the exogenous tobacco smoke component benzo[a]pyrene (BaP) and the persistent organic pollutant 2,3,7,8-Tetrachlorodibenzo-p-dioxin (TCDD) which induce ROS via AhR due to the enzymatic activity of its downstream marker CYP1A1 in NHEKs and skin^53–55^. This demonstrates again the ligand depending different effects of AhR activation^56–58^. Whether the antioxidant effects of the plant extract are responsible for the detected anti-inflammatory effects has to be determined in future studies.

The observed antioxidant potential of the plant extract may be based on two mechanisms: First, the antioxidant effects may be partly driven by direct radical scavenger effects of polyphenols^59^. Second, induction of antioxidant signaling pathways may play a role as preincubation of NHEKs with the extract could reduce the induction of ROS by H_2_O_2_, too. One important antioxidant signaling pathway is the Nrf2 pathway which is a key regulator of the antioxidant response in cells thus being able to reduce the ROS production caused by CYP1A1 metabolism. It was reported that the antioxidant character of tapinarof, coal tar and soybean tar glyteer are mainly based on Nrf2 activation^16,17,24^. Therefore, we measured the expression of NAD(P)H: quinone oxidoreductase 1 (NQO1), a downstream marker of Nrf2 which can eliminate reactive oxygen species^60^. The NQO1 expression was elevated by the plant extract suggesting the involvement of Nrf2, but further experiments are needed to confirm the activation of Nrf2.

The examination of plant extracts, with their wide variety of bioactive compounds, appears to be a promising approach for identifying novel compounds for the local treatment of AD. Many studies have demonstrated the positive effects of various plant compounds^61^. The comparison of these studies is challenging as the effects of these compounds are depending on the target cells as well as the concentration used. For example, quercetin protects rat hepatoma cells against H_2_O_2_-induced cytotoxicity and apoptosis at low concentrations, but at high concentrations, it induces cytotoxicity and DNA fragmentation itself^62^. The pleiotropic character of many plant polyphenols results in diverse activities complicating the clinical use by the risk of off-target effects^63^.

Using the entire plant extract in comparison to a single compound has the advantage of combining different bioactive substances which may act synergistically. In addition, the extraction is less complicated and cheaper than the isolation of a single compound^64^. But as a natural source, the exact composition of a plant extract strongly depends on the time and location of material collection as well as on the extraction parameters^65^. It is therefore of particular importance, to use standardized protocols for harvesting and extraction to gain a comparable extract composition.

In this study a mixture of apple, curly cale and green tea extract was used, as the same combination had shown beneficial effects in a clinical cohort study with AD patients, which forms the basis for the present investigation^28^. Therefore, the results are limited to the effects of the whole plant extract mixture. Consequently, it is not possible to assess the effects and influence of the individual extracts, nor to determine any potential synergistic or confounding mechanisms. Further studies will be needed to clarify the role of each individual extract and enhance understanding of the mechanistic basis.

Although our results are limited to 2D monolayer and 3D organotypic in vitro data they provide some more insight into the potential beneficial effects of the plant extract. Anti-*S. aureus* activity, AhR activation and antioxidant activity support the promising properties of the plant extract as topical treatment option in AD which has to be verified in future studies. Our study joins the list of reports highlighting the potential of plant-derived compounds for the topical treatment of complex skin diseases such as AD.

## Methods

### Plant extract

The used plant extract was composed of a mixture of curly kale extract (Anklam Extract GmbH, Anklam, Germany), green tea extract (Eurochem Feinchemie GmbH, Gröbenzell, Germany) and apple extract (Herbstreith & Fox KG, Neuenbürg/Württ, Germany) as described^28^. In short, apples, curly kale and green tea were dried and milled (particle seize < 0,2 mm). The dry plant material of apple, curly kale and green tea (5 g each) was extracted in 50% ethanol (48 h, 22 °C). After centrifugation (4500x*g*, 15 min) the collected supernatant was dried under vacuum at 45 °C to obtain the powdered extract. Each extract powder was then initially solved together in 50% EtOH (containing 100 mg/mL of each extract) and this extract mixture was further diluted 1:10 in cell culture medium to a stock solution of 5% EtOH in cell culture medium containing 10 mg/mL of each single extract. Different dilutions of the stock solution in cell culture medium were used for the stimulation experiments. Adequate dilutions of 5% EtOH in cell culture medium served as control vehicle for stimulation experiments.

### Antimicrobial assay

The effect of the plant extract mixture on three different *Staphylococcus (S.) aureus* strains, two clinical isolates from lesional skin of AD patients (*S. aureus* strain 178 P21 and *S. aureus* strain 185 P29, identity verified by MALDI-TOF mass spectrometry; MALDI Biotyper; Bruker, Billerica, MA, USA) and one ATCC strain (*S. aureus* ATCC 6538), were evaluated in an antimicrobial assay. In detail, 7 mL trypticase soy broth (TSB, Merck Millipore, Darmstadt, Germany) was inoculated with different strains of *S. aureus* from blood agar. The next day, 150 µL of this overnight culture was used to inoculate again 7 mL TSB. After approximately 2 h the bacteria were washed with phosphate-buffered saline (PBS, Thermo Fisher Scientific, Schwerte, Germany) and adjusted to an OD_600_ of 0.2 in cell culture medium (Dermalife Basal medium supplemented with 6 mM L-Glutamine) (CellSystems, Troisdorf, Germany). This bacteria solution was further diluted 1:2000 in 1 mL cell culture medium containing different dilutions of the plant extract or corresponding vehicle. The starting concentrations of the different *S. aureus* strains are shown in Fig. 1 (0 h) and ranged from ~ 10^4^ – 10^5^ CFUs/mL. After 2 h incubation without shaking at 37 °C, colony forming units (CFU) were determined by plating serial dilutions on TSB agar plates and counting after overnight incubation with the automated colony counter reader Synbiosis ProtoCOL SR (Synbiosis, Cambridge, UK).

### Determination of the effects of the plant extract on adherent and intracellular bacteria by intracellular infection assay

A modified lysostaphin protection assay was used to test if the plant extract mixture had an impact on the adherence of *S. aureus* to primary normal human epidermal keratinocytes (NHEKs, pooled from 4 donors, PromoCell, Heidelberg, Germany) or the number of intracellular living *S. aureus* in NHEKs. Post-confluent NHEKs seeded in 24-well plates were preincubated for 24 h with the plant extract mixture or vehicle (5% EtOH), both 1:800 diluted in cell culture medium. An *S. aureus* culture of an OD_600_ of 0.2 of the *S. aureus* strain 178 P21 was prepared in cell culture medium as described above. For bacterial treatment of the NHEKs, bacterial culture was further diluted 1:50 in cell culture medium containing plant extract or vehicle, both diluted 1:800. After exposure of NHEKs with the bacteria/plant extract mixture for 2 h, supernatant was harvested and plated on TSB agar for quantifying the number of extracellular bacteria. Then, PBS was added and the plate was intensively slewed 10 times horizontally and vertically before harvesting and plating the supernatant again (now containing the loosely attached bacteria). For determination of the number of strongly attached bacteria, first, the total number of strongly attached and intracellular bacteria was measured by adding 0.1% Triton-X 100 (Sigma-Aldrich Chemie GmbH, Taufkirchen, Germany) for 10 min at 37 °C to lyse NHEKs. Triton-X is a non-ionic detergent that disrupts membranes by interaction with the lipid bilayer. In comparison to eukaryotic cells that only have phospholipid bilayer membranes, bacteria have a peptidoglycan-based cell wall that is more protective against detergents^66^. Therefore, short treatment with the mild detergent 0.1% Triton-X is commonly used for the lysis of keratinocytes in intracellular infection assays^67^. To improve the lysis, cells were additionally sonicated using a sonicator (UP200St Hielscher, Hielscher Ultrasonic, Teltow, Germany), at 60% amplitude with eight pulses of 1 s with 3 s interval between pulses to prevent overheating. The lysate was plated on TSB agar plates. To determine the number of intracellular bacteria in parallel experiments, NHEKs were first incubated with lysostaphin (Sigma-Aldrich Chemie GmbH, Taufkirchen, Germany) (10 µg/mL) for 1 h to remove all attached bacteria. In contrast to the classical gentamycin protection assay, lysostaphin instead of gentamycin was used for extracellular bacterial killing as gentamycin is discussed to be internalized by the host cells and thus may have impact on the intracellular bacteria^68^. Afterwards, NHEKs were washed with PBS and then lysed with 0.1% Triton-X 100 for 10 min at 37 °C. The lysate was further sonicated as described and then plated on TSB agar plates. After overnight incubation, colonies were counted with Synbiosis ProtoCOL SR (Synbiosis, Cambridge, UK). The number of strongly attached bacteria was calculated by subtracting the number of intracellular bacteria from the number of both strongly attached and intracellular bacteria.

### Stimulation of NHEKs with living *S. aureus* for gene expression analysis

*S. aureus* can induce inflammation in keratinocytes. To check if the plant extract may inhibit the inflammatory response, stimulation experiments with living *S. aureus* were conducted: *S. aureus* culture of an OD_600_ of 0.2 for the two clinical isolates *S. aureus* strain 178 P21 and *S. aureus* strain 185 P29 were prepared in cell culture medium as described above. Then, bacteria were washed with PBS and diluted in cell culture medium to a concentration of 1*10^7^ CFU/mL. A 1:800 dilution of both the plant extract and the vehicle was prepared in cell culture medium. NHEKs, cultured in 24-well plates, were preincubated with vehicle for 24 h and then treated with the bacteria for 3 h in absence (*S. aureus*) or in presence of the extract (Simult. Inc. extract + *S. aureus*). NHEKs were also preincubated with the plant extract for 24 h and then treated with the bacteria in absence of the plant extract for 3 h (Preinc. Extract + *S. aureus*). Afterwards cells were washed with PBS and 200 µg/mL gentamycin (Carl Roth, Mannheim, Germany) was added. Gentamycin was added to inhibit an overgrowth of bacteria, which would kill the NHEKs. After 20 h, cells were washed with PBS and RNA was isolated.

### Stimulation of NHEKs with AD cytokines and the plant extract

For stimulation experiments, NHEKs were seeded in a 24-well plate in Dermalife Complete medium (CellSystems, Troisdorf, Germany). After reaching 90–100% confluency, cells were incubated with medium containing 1.3 mM CaCl_2_ for 48 h to induce differentiation. Differentiated NHEKs were stimulated with a cytokine mixture (IL-4, IL-13, TNFα and IL-22 (each 10 ng/mL) to mimic an AD-like inflammation. Simultaneously, these cells were incubated with the plant extract mixture or vehicle, both 1:800 diluted, for 20 h at 37 °C, at 5% $\:{\text{C}\text{O}}_{2}$ atmosphere. Subsequently, cells were washed with PBS and RNA was isolated.

### Stimulation of an organotypic three-dimensional (3D) skin equivalent

An organotypic three-dimensional (3D) skin equivalent was generated as previously described^69^ with some minor modifications: First, keratinocytes were isolated from the epidermis of abdominal human skin. The use of ex vivo skin for isolation of keratinocytes was approved by the Local Ethics Committee of the Medical Faculty, Kiel University (Kiel, Germany) (D 603/22) in accordance with the Declaration of Helsinki Principles guidelines. Secondly, Dermalife medium was used for seeding keratinocytes and instead of KGM for the serum-free keratinocyte differentiation medium (SKDM). Skin equivalents were treated with SKDM medium containing the AD cytokine mixture (IL-4, IL-13, TNFα and IL-22; each 10 ng/mL) and the vehicle or plant extract (1:200 diluted in medium), respectively. After 20 h, two 6 mm biopsies were taken from each skin equivalent sample. One biopsy was used for RNA isolation and the other was embedded in paraffin for histological examinations.

### RNA isolation and real-time PCR

After stimulation, gene expression levels of different AD-relevant genes were measured via real-time PCR. Therefore, RNA of the keratinocytes and the 3D skin equivalents were isolated using the Crystal RNAmagic kit (Bio-lab products, Gödenstorf, Germany) and transcribed into complementary DNA (cDNA) using Prime-Script reverse transcriptase kit (Takara Bio, Saint-Germain-en-Laye, France). The cDNA was used as template in a real-time PCR utilizing the QuantStudio3 System (Thermo Fisher Scientific, Schwerte, Germany), following the temperature profile outlined in prior descriptions^70^. Gene expression levels of carbonic anhydrase II (CA2), CC-motif chemokine ligand 5 (CCL5), CC-motif chemokine ligand 26 (CCL26), Cytochrome P450, Family 1, Subfamily A Member 1 (CYP1A1), filaggrin (FLG), interleukin-8 (IL-8, *CXCL8*), interleukin-6 (IL-6), NAD(P)H quinone dehydrogenase 1 (NQO1), homo sapiens ribosomal protein L38 (RPL38) and tumor necrosis factor alpha (TNFα) were measured. All used primers were intron-spanning and listed in Table 1. The gene expression levels were normalized to the constitutively expressed ribosomal protein L38 (*RPL38*) gene.

### Immunohistochemistry

Formalin-fixed paraffin-embedded 3D skin equivalents were stained with anti-CYP1A1 mouse antibody (Santa Cruz, CYP1A1 (B-4), sc-25304), 1:50 diluted in Tris buffered saline/1% BSA. Biotinylated goat anti-mouse antibody was used as secondary antibody (Vector Laboratories; Ref. BA-9200, Biozol, Eching, Germany), 1:300 diluted in Tris-buffered saline/1% BSA. For detection of biotinylated molecules, the immunoperoxidase detection system Vectastain ABC reagent and Vector Nova Red-Substrate (Vector Laboratories Biozol, Eching, Germany) were used.

**Table 1:** List of primers used for gene expression analysis via real-time PCR.

Gene	Forward primer	Reverse primer
Carbonic Anhydrase II, CA2	5′-AACAATGGTCATGCTTTCAACG-3′	5′-TGTCCATCAAGTGAACCCCAG-3′
CC motif chemokine ligand 5, CCL5	5’- TATTCCTCGGACACCACACC-3‘	5’-GTGACAAAGACGACTGCTGG-3’
CC-motif chemokine ligand 26, CCL26,	5′-AATTGAGGCTGAGCCAAAGA-3′	5′-ATCAGGCCCTTCTCAGGTTT-3′
Cytochrome P450 Family 1 Subfamily A Member 1, CYP1A1	5’- CACCATCCCCCACAGCAC-3’	5’-ACAAAGACACAACGCCCCTT-3’
Filaggrin, FLG	5′-GGCAAATCCTGAAGAATCCAGATG-3′	5′-GGTAAATTCTCTTTTCTGGTAGACTC-3′
Interleukin 6, IL6	5’-GGTACATCCTCGACGGCATCT-3’	5’-GTGCCTCTTTGCTGCTTTCAC-3’
Interleukin 8, CXCL8	5’-TCCTGATTTCTGCAGCTCTGT-3’	5’-AAATTTGGGGTGGAAAGGTT-3’
NAD(P)H quinone dehydrogenase 1, NQO1	5’-AGCTCACCGAGAGCCTAGTT-3’	5’-GTGCTCTTCTGCCGACCAT-3’
Homo sapiens ribosomal protein L38, RPL38	5′-TCAAGGACTTCCTGCTCACA-3′	5′-AAAGGTATCTGCTGCATCGAA-3′
Tumor necrosis factor alpha, TNFα	5’-CCTGCTGCACTTTGGAGTG-3’	5’-GCTTGAGGGTTTGCTACAACA-3’

### Measurement of AhR activation via AhR gene luciferase activity assay

For determination of AhR activation, NHEKs were transfected by using the FugGENE HD transfection reagent (Promega, Madison, WI, USA) with the pGudLuc6.1 reporter plasmid (generously gifted by M. Denison, U.C. Davis) (300 ng/well) in which the *firefly* luciferase gene expression is dependent on AhR activation. The co-transfected pGL4.74 *renilla* luciferase plasmid [hRluc/TK] (Promega, Madison, WI, USA) (30 ng/well) functioned as reference plasmid to control transfection efficiency. Medium was changed 6 h after transfection. One day after transfection, cells were stimulated with the AD cytokine mixture (IL-4, IL-13, TNFα and IL-22; each 10 ng/mL) with the plant extract or vehicle for 24 h as described above. Luciferase activity was determined by following the protocol of *firefly*/*renilla* Dual Luciferase activity assay (Sigma-Aldrich, St Louis, MO, USA). AhR activation was calculated as the ratio of *firefly* to *renilla* luciferase activities in each sample.

### AhR knockdown by siRNA and AhR inhibition

AhR knockdown and AhR inhibition experiments were conducted to determine the role of AhR. For the AhR knockdown experiments, 70% confluent NHEKs were transfected with 5 nM AhR siRNA (AHR Silencer^®^select siRNA S1199, Ambion) or nonsilencing control siRNA (Silencer^®^select negative control siRNA, Ambion) using 1 µL HiPerfect transfection reagent (Qiagen, Hilden, Germany) per well. After 24 h medium was changed. When NHEKs reached 100% confluency, cells were stimulated with the AD cytokines and plant extract as described above and RNA was isolated.

For AhR inhibition, CaCl_2_-differentiated NHEKs were preincubated with 10 µM AhR inhibitor CH-223191 (Cayman Chemicals, Ann Arbor, MN, USA) for 1 h. Then, cells were stimulated in presence of 10 µM CH-223191 with the plant extract and cytokines as described and RNA was isolated. 0.1% DMSO functioned as control.

### Measurement of intracellular reactive oxygen species

Intracellular reactive oxygen species were measured using the DCFDA/H2DCFDA - Cellular ROS Assay Kit (abcam, Cambridge, UK). Therefore, NHEKs were seeded in a 96-well plate. When reaching 100% confluency, cells were stimulated with the AD cytokine mixture (IL-4, IL-13, TNFα and IL-22; each 10 ng/mL) with vehicle or the plant extract for 24 h, both 1:800 diluted. After stimulation, cells were washed with PBS and stained using the DCFDA assay as described in the manual. For determination of preventive effects of the plant extract, cells were preincubated with the plant extract for 24 h. Then, cells were washed with PBS and stained as described. After 45 min, medium with 250 µM H_2_O_2_ (Sigma-Aldrich Chemie GmbH, Taufkirchen, Germany) was added for 10 min. Fluorescence was measured in a plate reader (Infinite M Plex, Tecan, Crailsheim, Germany) with extinction wavelength of 485 nm and emission wavelengths of 535 nm.

### Statistics

The software GraphPad prism 6 was used for statistical analysis (GraphPad Software, San Diego, CA, USA). Normality was analyzed by D’Agostino-Pearson normality test. Statistical significances of parametric datasets were tested by One Way ANOVA with subsequent Bonferroni multiple comparison test. Unpaired t-test was used for the comparison of two groups, also for small sample sizes (*n* < 5)^71^. In case of nonparametric datasets, Kruskal-Wallis test with subsequent Dunn’s multiple comparison test or Mann Whitney tests for the comparison of two groups were used.

## Electronic supplementary material

Below is the link to the electronic supplementary material.

Supplementary Material 1

## Data Availability

The datasets generated during the current study are available from the corresponding author on reasonable request.
