# Supplementary material for: Atopic dermatitis-related anti-inflammatory in vitro effects of a plant extract mixture
Source: Sci Rep. 2025 Jul 3;15:23828. doi: 10.1038/s41598-025-09053-4 (PMC12229520; doi:10.1038/s41598-025-09053-4)
Supplement: Supplementary file 1 — Supplementary Material 1 [file 41598_2025_9053_MOESM1_ESM.pdf]

Supplementary Information for:

Atopic dermatitis-related anti-inflammatory in vitro effects of a plant extract mixture

Authors: Nina Heinemann[1], Franziska Rademacher[1], Henning Vollert[2], Regine Gläser[1], Jürgen Harder[1*]

[1] Department of Dermatology, Kiel University, Kiel, Germany

[2] Bioactive Food GmbH, Bad Segeberg, Germany

This file includes:

- Supplementary Figures S1-S4

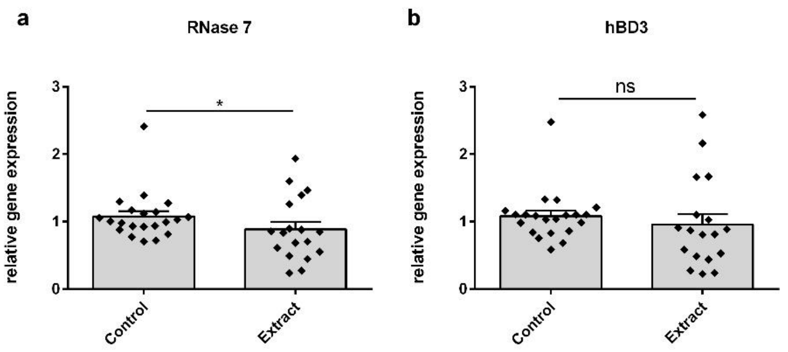

Supplementary Figure S1: Incubation with Plant Extract Mixture Did Not Induce Principal *S. aureus*-Killing Antimicrobial Peptides. $CaCl_2$-differentiated NHEKs were left unstimulated (Control) or stimulated with the plant extract (Extract) for 20 h. Gene expression levels of (**a**) RNase 7 and (**b**) hBD3 were determined by real-time PCR. Statistical significance was tested by Mann-Whitney test (n=18 *p < 0.05, ns = not significant). Bars indicate means + SEM.

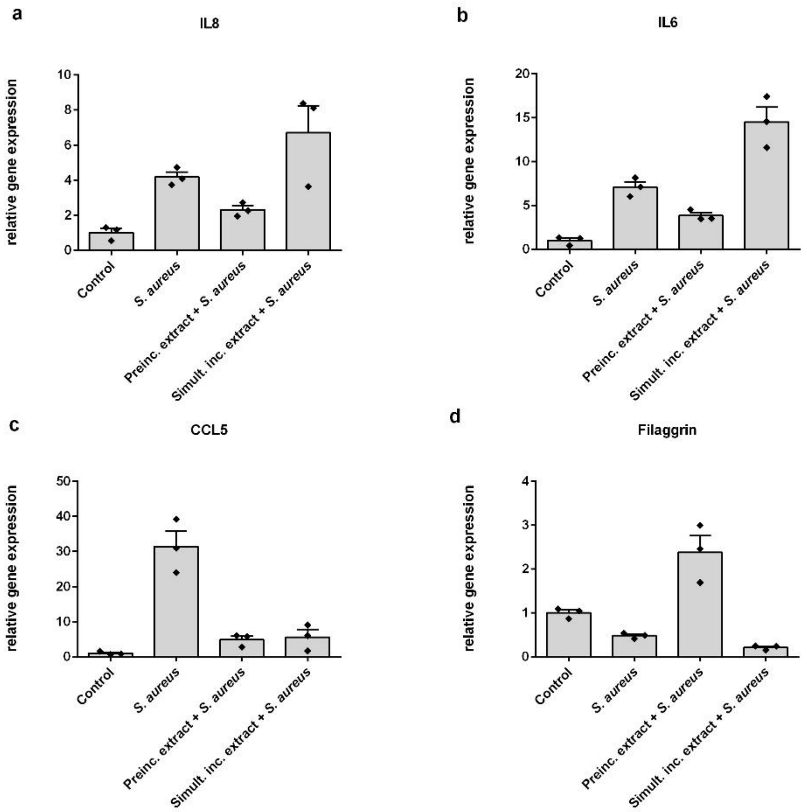

Supplementary Figure S2: Preincubation with Plant Extract Mixture Reduced Inflammtion Induced by *S. aureus* 185 P29. NHEKs were preincubated with the plant extract for 24 h and subsequently treated with the *S. aureus* strain 185 P29 for 3 h, followed by gentamycin addition and further incubation for 24 h. Gene expression levels of (**a**) IL8, (**b**) IL6, (**c**) CCL5 and (**d**) filaggrin were determined by real-time PCR. The data shown are from one experiment performed in triplicate stimulations.

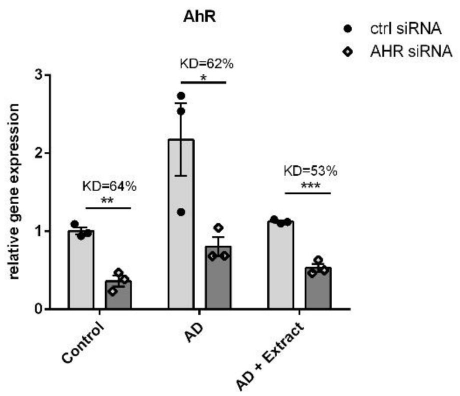

Supplementary Figure S3: AhR Knockdown Efficiency. NHEKs were transfected with AhR siRNA or control siRNA. 48 h our later, NHEKs were stimulated with the AD-associated cytokine mixture (IL-13, IL-4, IL-22 and TNF-alpha, each 10 ng/mL) either in the absence (AD) or in the presence of the plant extract (AD + Extract). The knockdown efficiency (KD) was indicated in percent above the corresponding bars. Gene expression levels of AhR were determined by real-time PCR. Statistical significance was tested by unpaired t-test (n=3; *p < 0.05; **p < 0.01; ***p < 0.01; ns = not significant). Bars indicate means + SEM**.**

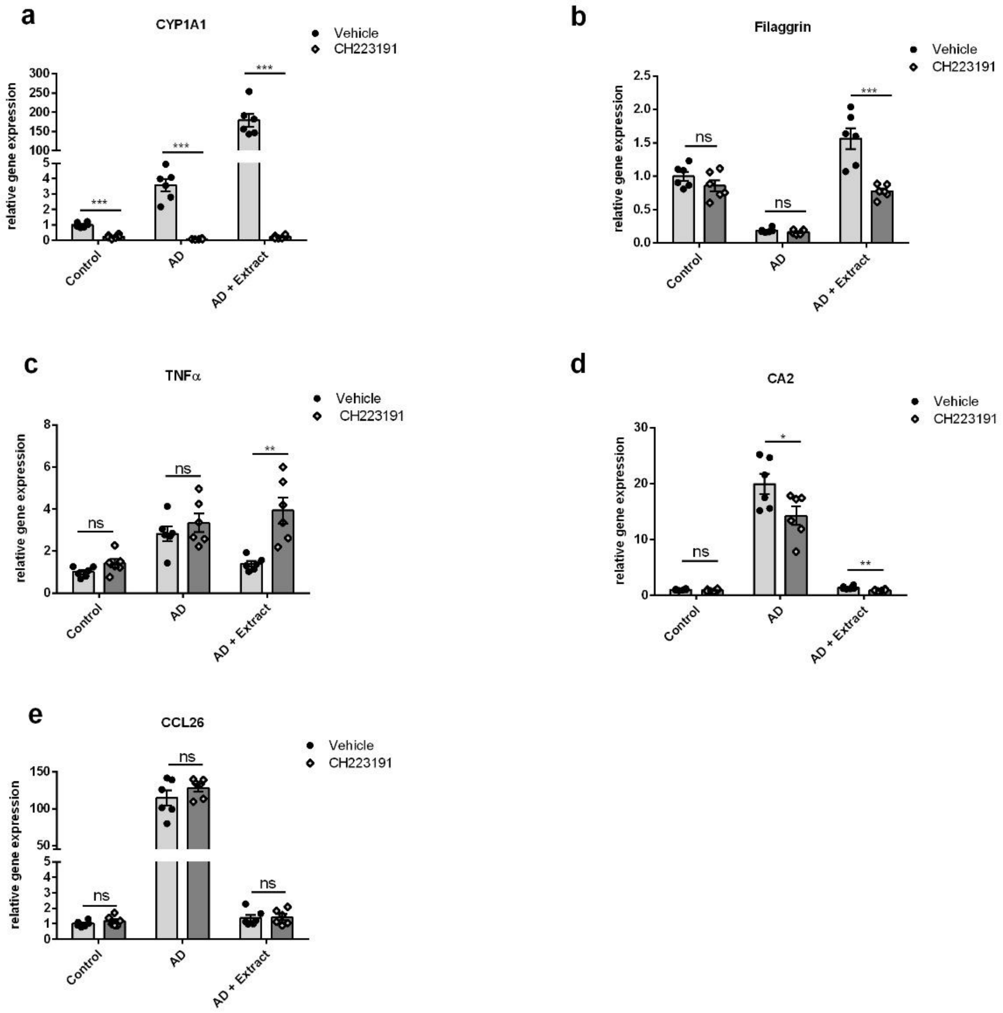

Supplementary Figure S4: Impact of AhR Inhibition on the Effects of the Plant Extract Mixture in the 2D AD Model. $CaCl_2$-differentiated NHEKs were preincuabted with 10 µM AhR inhibitor CH223191 or vehicle for 1 h. Then, NHEKs were stimulated in the presence of 10 µM CH223191 with the AD-associated cytokine mixture (IL-13, IL-4, IL-22 and TNF-alpha, each 10 ng/mL) either in the absence (AD) or in the presence of the plant extract (AD + Extract). Gene expression levels of (**a**) CYP1A1 (**b**) filaggrin (**c**) TNFα (**d**) CA2 and (**e**) CCL26 were determined by real-time PCR. Statistical significance was tested by unpaired t-test (n=6; *p < 0.05; **p < 0.01; ***p < 0.001; ns = not significant). Bars indicate means + SEM.
